# Developmental Considerations of Sperm Protein 17 Gene Expression in Rheumatoid Arthritis Synoviocytes

**DOI:** 10.1080/1044667021000095186

**Published:** 2002-06

**Authors:** Yuichi Takeoka, Thomas P. Kenny, Hisashi Yago, Mitsuru Naiki, M. Eric Gershwin, Dick L. Robbins

**Affiliations:** 1 Institute of Bio-Active Science Nippon Zoki Pharmaceuticals, Ltd. Hyogo Japan; 2 Division of Rheumatology/Allergy and Clinical Immunology Department of Internal Medicine University of California at Davis Davis CA 95616 USA

## Abstract

Rheumatoid arthritis (RA) is an autoimmune disease characterized by proliferative synovial tissue. We used mRNA differential display and library subtraction to compare mRNA expression in RA and osteoarthritis (OA) synoviocytes. We initially compared the mRNA expression patterns in 1 female RA and 1 OA synovia and found a differentially expressed 350 bp transcript in the RA synoviocytes which was, by sequence analysis, 100% homologous to sperm protein 17 (Sp17). Moreover, the Sp17 transcript was found differentially expressed in a RA synovial library that was subtracted with an OA synovial library. Using specific primers for full length Sp17, a 1.1 kb transcript was amplified from the synoviocytes of 7 additional female RA patients, sequenced and found to 100% homologous to Sp17. Thus, we found the unexpected expression of Sp17, a thought to be gamete-specific protein, in the synoviocytes of 8/8 female RA patients in contrast to control OA synoviocytes. Interestingly, Sp17's structural relationship with cell-binding and recognition proteins, suggests that Sp17 may function in cell-cell recognition and signaling in the RA synoviocyte. Further, Sp17 could have a significant regulatory role in RA synoviocyte gene transcription and/or signal transduction. Thus, Sp17 could have an important role in RA synoviocyte proliferation or defective apoptosis. Finally, the presence of Sp17 in synoviocytes has interesting developmental considerations.


*Developmental Immunology*, 2002 Vol. 9 (2), pp. 97–102

Taylor & Francis healthsciences

### Developmental Considerations of Sperm Protein 17 Gene Expression in Rheumatoid Arthritis Synoviocytes

YUICHI TAKEOKA[a,b], THOMAS P. KENNY[b], HISASHI YAGO[a], MITSURU NAIKI[a], M. ERIC GERSHWIN[b] and DICK L. ROBBINS[b,*]

[a]*Institute of Bio-Active Science, Nippon Zoki Pharmaceuticals, Ltd., Hyogo, Japan;* [b]*Division of Rheumatology/Allergy and Clinical Immunology, Department of Internal Medicine, University of California at Davis, Davis, CA 95616, USA*

Rheumatoid arthritis (RA) is an autoimmune disease characterized by proliferative synovial tissue. We used mRNA differential display and library subtraction to compare mRNA expression in RA and osteoarthritis (OA) synoviocytes. We initially compared the mRNA expression patterns in 1 female RA and 1 OA synovia and found a differentially expressed 350 bp transcript in the RA synoviocytes which was, by sequence analysis, 100% homologous to sperm protein 17 (Sp17). Moreover, the Sp17 transcript was found differentially expressed in a RA synovial library that was subtracted with an OA synovial library. Using specific primers for full length Sp17, a 1.1 kb transcript was amplified from the synoviocytes of 7 additional female RA patients, sequenced and found to 100% homologous to Sp17. Thus, we found the unexpected expression of Sp17, a thought to be gamete-specific protein, in the synoviocytes of 8/8 female RA patients in contrast to control OA synoviocytes. Interestingly, Sp17's structural relationship with cell-binding and recognition proteins, suggests that Sp17 may function in cell–cell recognition and signaling in the RA synoviocyte. Further, Sp17 could have a significant regulatory role in RA synoviocyte gene transcription and/or signal transduction. Thus, Sp17 could have an important role in RA synoviocyte proliferation or defective apoptosis. Finally, the presence of Sp17 in synoviocytes has interesting developmental considerations.

*Keywords*: Gene expression; Rheumatoid arthritis; Sperm protein 17; Developmental biology

### INTRODUCTION

Rheumatoid arthritis (RA) is an autoimmune disease characterized by proliferative synovial tissue, including proliferating synoviocytes and subsynovial tissue infiltrated with lymphoid and other immune cells. However, our current understanding of the pathogenic mechanisms operative in RA synovitis is incomplete. T cells, B cells, macrophages, cytokines, MHC antigens and other gene products are important in RA as is synoviocyte proliferation and the subsequent destruction of articular cartilage and bone (Goronzy and Weyand, 1997; Imamura *et al.*, 1998). Understanding the mechanisms responsible for synoviocyte hyperplasia, e.g. proliferation versus defective apoptosis, is necessary to better understand RA.

Sperm protein (Sp17) is a highly conserved and autoantigenic protein that was originally cloned, sequenced and characterized as the 17 kD member of the rabbit sperm autoantigen family of Sp (O'Rand *et al.*, 1988; Richardson *et al.*, 1994). Sp17 functions in the binding of sperm to zona pellucida (O'Rand and Widgren, 1994). The Sp17 gene has also been found in the mouse, rat, monkey, dog, baboon and humans (Lea *et al.*, 1996) and data initially indicated that Sp17 was testes-specific by Northern blot analysis and localized in the cytoplasm of spermatogenic cells of testes (Adoyo *et al.*, 1997). However, recently Sp17 has been found in some lymphoid tissue and certain malignancies (Dong *et al.*, 1997), suggesting that Sp17 belongs to a new family of molecules, the cancer-testes antigen (CTS).

Sp17 has a peptide structure which shares homology and motifs with cell-junctional proteins. The motifs of Sp17 include: a protein kinase A region, a sulfate binding region, and a calmodulin binding site (Yamasaki *et al.*, 1995). Thus, Sp17 may have an important role in uncontrolled cell proliferation.

*Corresponding author. Tel.: +1-530-752-2884. Fax: +1-530-754-6047. E-mail: dlrobbins@ucdavis.edu

ISSN 1044-6672 print © 2002 Taylor & Francis Ltd
DOI: 10.1080/1044667021000095186

Because RA synovium is characteristically proliferative, we compared the mRNA expression patterns in RA and osteoarthritis (OA) synoviocytes using differential display and library subtraction. Interestingly, we found a differentially expressed 350 base pair transcript in the RA synoviocytes which was 100% homologous to Sp17. Moreover, we demonstrated the highly unexpected differential mRNA expression of Sp17 in the synoviocytes of 8/8 female RA patients. Further, indomethacin and Neurotropin, which has been reported to have beneficial effects in animals with immune abnormality (Naiki *et al.*, 1989; 1991; Kato *et al.*, 1991), inhibited Sp17 gene expression in RA synoviocytes, whereas dexamethasone had no effect on Sp17 gene expression in RA synoviocytes.

### MATERIALS AND METHODS

### Preparation of Synovial Cells (SC)

Synovial tissue was obtained incidental to clinically indicated surgical procedures (synovectomy, total joint replacement) on seropositive RA patients, as previously described (Ermel *et al.*, 1997). Human synovium was cut into small 5-mm pieces and digested with collagenase (EC 3.4.24.3; Type IA, Sigma Chemical Co., St. Louis, MO, USA) and deoxyribonuclease I (EC 3.1.21.1; DNase I Type IV, Sigma Chemical). Single cells were decanted from the undigested material and washed in Hank's balanced salt solution (HBSS) (JRH Laboratory, Woodland, CA, USA). Synovial cells (SC) were resuspended in Dulbecco's modified MEM.F12 (D/F12) medium (JRH Laboratory) containing 10% fetal calf serum (FCS) and gentamicin (Sigma Chemical) to 5000 cells/ml and 24 well plates (Corning, Corning, NY, USA) were seeded with 2 ml of SC. Seeded and incubated SC were trypsinized and passed. After two or three passages, the cultured RA-SC were fibroblast-like SC. Eight RA-SC lines were isolated from 8 different female synovia.

### Agents

Dexamethasone (dexamethasone–water soluble) and indomethacin were obtained from Sigma Chemical, St. Louis, MO. Indomethacin was dissolved in ethanol and diluted with the medium as described later. Ethanol was used at 0.005% in the culture medium. Dexamethasone and indomethacin were used at concentrations of $10^{-6}$ M. Neurotropin™, which is a unique non-protein extract isolated from a inflamed dermis of rabbits inoculated with vaccinia virus, was obtained from Nippon Zoki Pharmaceutical Co., Ltd. (Osaka, Japan). It has been also clinically used as an analgesic and as an anti-allergy drug with few side effects in humans. Neurotropin was diluted with medium at concentrations of 0.1 or 0.01 Neurotropin unit/ml.

### Cell Culture

Wells were allowed to grow to 75% confluence. Passed and incubated SC were washed three times with HBSS. One ml of D/F12 medium without FCS was pipetted into each well, and cultured for 1 day to synchronize the cell cycle. The medium was changed to D/F12 medium with 10% of FCS. At this time, $10^{-6}$ M of dexamethasone or indomethacin, or 0.1 or 0.01 unit/ml of Neurotropin was added to the wells. Saline was diluted 1:1000 in culture medium as a control. The control for indomethacin contained 0.005% of ethanol in culture medium as mentioned above. The cells were harvested after culture for 3 days.

### RNA Preparation and Removal of DNA Contamination from RNA

At the desired time points, the cells were washed twice with PBS, and 1.0 ml of Isogen (Wako Chemical Industry, Osaka, Japan) was added directly to the cells for the isolation of total RNA. Total RNA was prepared from the cultured cells using the acid guanidine thiocyanate-phenol/chloroform method.

Total cell RNA (50 μg) was treated with 10 units of DNase I to remove chromosomal DNA contaminants from the RNA sample obtained by using the protocols from GenHunter (Message Clean kit; GenHunter, Brookline, MA, USA). After extraction with phenol/chloroform and ethanol precipitation with 3 M NaOAC, the RNA was redissolved in DEPC-treated water. The redissolved RNA samples were quantitated by OD260 after 1:1000 dilution, and purified RNA samples (1–2 μg/μl) were stocked at −80°C until ready for use.

### mRNA Differential Display

Purified RNA samples (0.1 μg/μl) were reverse-transcribed with one of the one-base-anchored H-T11G primer (5′-AAGCTTTTTTTTTTTG-3′) using MMLV reverse transcriptase according to the manufacturer's instructions (RNAimage: GenHunter). The reverse-transcribed cDNA was PCR-amplified in combination with the 5″ arbitrary primer (5′-AAGCTTCATTCCG-3′) and the H-T11G primer. The cycling parameters were as follows: 94°C for 30 s, 45°C for 2 min, 72°C for 30 s for 40 cycles, followed by 72°C for 5 min. The cDNAs were labeled with [$^{35}$S]-dATP (NEN, Boston, MA, USA). The amplified cDNAs were separated on a 6% acrylamide sequencing gel. [$^{35}$S] labeled cDNAs were exposed to X-ray film (Fuji, Tokyo, Japan).

Differentially displayed cDNAs were excised from the dried sequencing gel, recovered and reamplified in a reaction volume of 40 μl for 40 cycles under the same conditions mentioned above. Reamplified PCR products were cloned into the PCR II vector. DNA sequence analysis was carried out using Taq DyeDeoxy Terminator Cycle Sequencing Kit (Applied Biosystems, Foster City,

CA, USA). The samples were sequenced on the ABI373A DNA sequencer (Applied Biosystems) and were automatically recorded and analyzed using Genescan 672 software.

### RNA-based Primed PCR

RA-SC were harvested for RNA extraction when cultures reached about 75% confluence. Total RNAs were prepared by Isogen (Wako Chemical Industry). The purified RNA from RA-SC were reverse transcribed using either specific primers for Sp17 5′UTR (5′-GATGTGGTCAAGGAGCGAATC-3′) or CDS (5′-ATTCTGAGAGAGCAACCGGAC-3′) and subsequently amplified by PCR using either specific primer combinations for Sp17 5′UTR (5′-GATGTGGTCAAGGAGCGAATC and 3′-GCAGTCAGACACACTTGAC), or CDS (5′-ATTCTGAGAGAGCAACCGGAC and 3′-GAGTCTAAGATGGTGACTGATG). The PCR products were separated by 1.5% agarose gel with ethidium bromide, and were detected under ultra violet.

### RESULTS

### mRNA Differential Display of RA and OA Synoviocytes and Identification of Sp17

We initially found the differential expression of Sp17 mRNA using differential display and confirmed by library subtraction between a female RA patient's and an OA patient's synoviocytes. Figure 1 shows the sequence homology of the amplified transcript, we designated Yu 41, and it's best match to gene sequences searched using Genbank. There was a 99.43% homology to the 3′ coding region and UTR of human Sp17. Because of the size of the band amplified by differential display, we were only able to sequence the 3′ end of the Sp17 gene. Therefore, we then

FIGURE 1 Homology of the amplified and sequenced cDNA transcript, Yu 41, to Sp17, the best match in Genbank.

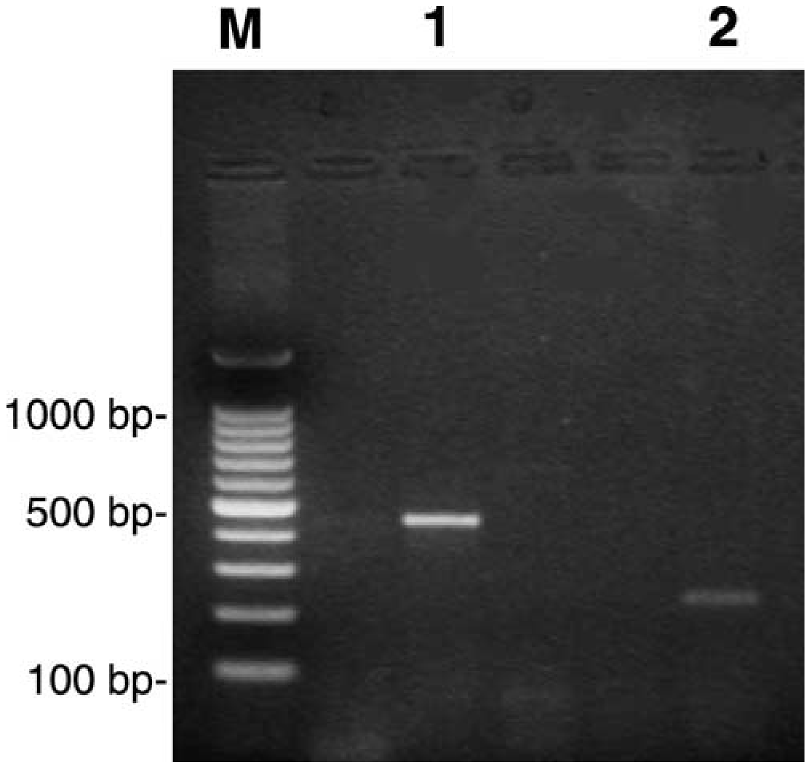

FIGURE 2 This figure shows the respective bands amplified using specific primers from RA synviocytes. (A) *Lane 1*: 1.6 5′UTR 5′-GATGTGGTCAAGGAGCGAATC; 3′-GCAGTCACTGACACACTTGAC. (B) *Lane 2*: CDS, 5′-ATTCTGAGAGAGCAACCGGAC; 3′-GAGTCTAAGATGGTGACTGATG. Analysis indicated that the band in lane 1 was 100% homologous with the 5′ UTR and the band in lane 2 homologous with the coding region of Sp17. Lane M is the marker.

designed specific 5′ and coding region primers to amplify the remainder of the Sp17 gene from the RA synoviocytes. Figure 2 shows the respective bands amplified. Sequence analysis indicated that the band in lane 1 was 100% homologous with the 5′ UTR and the band in lane 2 with the coding region of Sp17.

We then examined synoviocytes obtained from eight female RA patients and one additional OA patient by PCR amplification using our Sp17 specific primers. All 8 of the RA patients synoviocytes were strongly positive for Sp17 gene expression. Although PCR amplification of Sp17 in the OA patient's synoviocytes showed the presence of the Sp17 gene expression, it was barely positive (data not shown).

### Sp17 Expression and Modulation in RA Synoviocytes

We next examined modulation of Sp17 gene expression in RA synoviocytes by dexamethasone, indomethacin, and Neurotropin. Figure 3 is a representative example of such modulation. Lane 1 shows control RA synoviocyte Sp17 expression. Lane 2 shows the effect of 0.005% ethanol, as a control, which was used to solubilize indomethacin. Lanes 3 and 4 show the inhibition effect of different concentrations of Neurotropin on Sp17 gene expression while Lane 5 shows the inhibitory effect of indomethacin, and Lane 6 shows the lack of inhibition by dexamethasone. Thus, both Neurotropin and indomethacin inhibited Sp17 gene expression in RA synoviocytes but dexamethasone did not.

### DISCUSSION

Sp17 is a highly conserved autoantigenic protein that was originally described as the 17 kDa member of the rabbit sperm autoantigen family of sperm proteins. Sp17 was cloned, sequenced and characterized in the rabbit as an autoantigen that functions in the binding of sperm to zona pellucida (O'Rand *et al.*, 1988; O'Rand and Widgren, 1994; Richardson *et al.*, 1994;). The Sp17 gene is also found in the mouse, rat, monkey, dog, baboon and humans but not in chicken or yeast (Lea *et al.*, 1996). Data initially indicated that Sp17 was testes-specific by Northern blot analysis and is localized in the cytoplasm of resting spermatogenic cells of the testes (Adoyo *et al.*, 1997). More recently, however, the Sp17 gene was found in sheep mucosa associated lymphoid tissue, the metastatic stage of a murine squamous cell carcinoma model, multiple myeloma and malignant lymphocytes (Dong *et al.*, 1997). Thus, it is likely that Sp17 is a member of a new family of molecules, CTS. These observations, coupled with the molecular structure of Sp17, suggest an important role in unregulated cell proliferation.

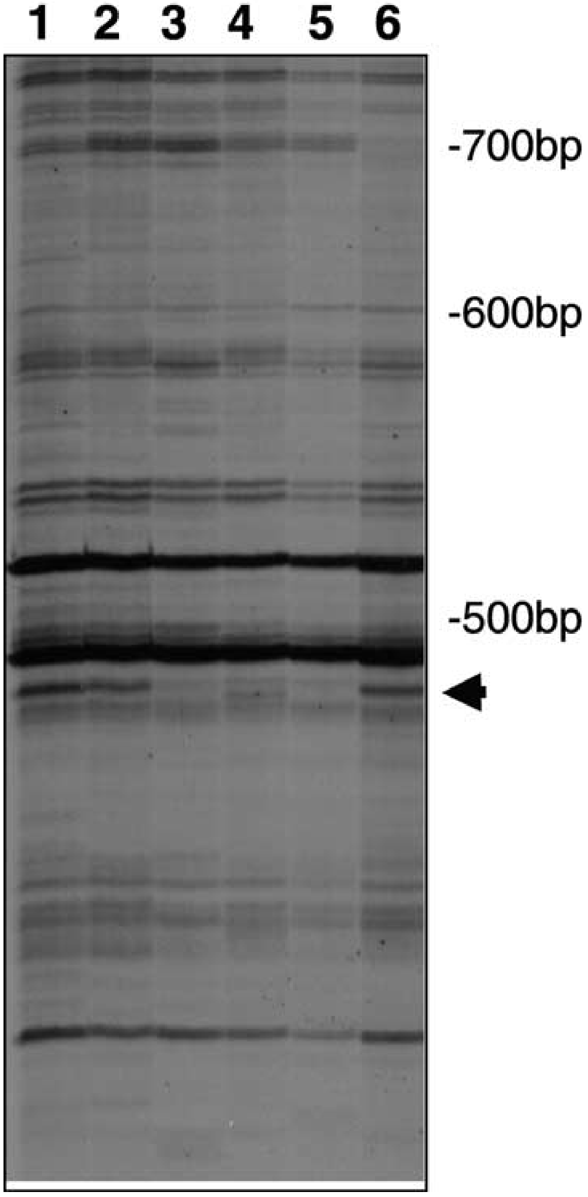

FIGURE 3 Modulation of RA SC mRNA Sp17 expression by Neurotropin (*Lane* 3, 0.1 nu/ml, *Lane 4*, 0.01 nu/ml), *Lane 5* is indomethacin ($10^{-6}$ molar with 0.005% of ethanol) and *Lane 6* is dexamethasone ($10^{-6}$ molar). *Lane 1* is control RA SC. *Lane 2* is a control of 0.005% ethanol used to solubilize indomethacin. The triangular arrow points to the Sp17 band in all lanes.

It is of interest that although dexamethasone showed no effect on Sp17 gene expression in RA synoviocytes, both indomethacin and Neurotropin inhibited expression of the Sp17 gene. The effect of Neurotropin is unknown but may be able to improve abnormalities autoimmune murine models (Naiki *et al.*, 1989; 1991; Kato *et al.*, 1991) and kallikreinkinin-fibrinolytic cascades in inflammation (Nishikawa *et al.*, 1992). On the other hand, it is well established that indomethacin and other NSAIDs inhibit gene expression in certain cancer cells such as colorectal and lung cancer (Pan *et al.*, 2001; Husain *et al.*, 2002), and the egr-1 gene in endothelial cells which promotes angiogenesis (Szabo *et al.*, 2001). It is, therefore, not surprising that indomethacin inhibited Sp17 expression in RA SC since they and cancer cells have in common, unregulated proliferation.

At this time we do not know whether, Sp17 has a functional role in the RA synoviocyte or whether, it is merely an embryonal antigen. However, the molecular structure of Sp17 strongly implies a functional role. In particular, the cAMP dependent protein kinase II alpha site suggests that Sp17 could be involved in regulation of gene transcription within the synoviocyte. This could have significant pathogenic effects if Sp17 or a gene it regulates were important in regulating proliferation or apoptosis. Moreover, the protein kinase alpha site could also be important in modulating signal transduction within the RA synoviocyte. Furthermore, the sulfate binding site could be important in binding polysaccharides produced within the RA synovium and affect their function. Finally, the calmodulin binding site could also allow Sp17 to contribute to signal transduction within the RA synoviocyte.

Therefore, although the function of Sp17 in the RA synoviocyte is unknown, the molecular structure strongly suggests that Sp17 may be linked to cell–cell communication, signal transduction, protein synthesis, regulation of gene transcription, proliferation and/or defective apoptosis. Further studies are indicated to better define the function of Sp17 in the RA synovium. Moreover, this study demonstrates the usefulness of mRNA differential display to detect altered gene expression in RA and other autoimmune diseases, and the potential usefulness of this approach to identify candidate genes as chemotherapeutic markers and targets.

### *Acknowledgements*

We greatly appreciate the efforts of our orthopedic colleagues to provide us with synovial tissue. Supported in part by the AMA-ERF.

### *References*

Adoyo, P.A., Lea, I.A., Richardson, R.T., Widgren, E.E. and O'Rand, M.G. (1997) "Sequence and characterization of the sperm protein Sp17 from the baboon", *Mol. Reprod. Dev.* **47**, 66–71.

Dong, G., Loukinova, E., Smith, C.W., Chen, Z. and Van Waes, C. (1997) "Genes differentially expressed with malignant transformation and metastatic tumor progression of murine squamous cell carcinoma", *J. Cell Biochem.* **Suppl. 29**, 90–100.

Ermel, R.W., Kenny, T.P., Wong, A., Chen, P.P., Malyj, W. and Robbins, D.L. (1997) "Analysis of the molecular basis of synovial rheumatoid factors in rheumatoid arthritis", *Clin. Immunol. Immunopathol.* **84**, 307–317.

Goronzy, J.J. and Weyand, C.M. (1997) "Rheumatoid arthritis: epidemiology, pathology and pathogenesis", *Primer Rheum. Dis.* **11**, 155–160.

Husain, S.S., Szabo, I.L. and Tamawski, A.S. (2002) "NSAID inhibition of GI cancer growth: clinical implications and molecular mechanisms of action", *Am. J. Gastroenterol.* **97**, 542–553.

Imamura, F., Aono, H., Hasunuma, T., Sumida, T., Tateishi, H., Maruo, S. and Nishioka, K. (1998) "Monoclonal expansion of synoviocytes in rheumatoid arthritis", *Arthritis Rheum.* **41**, 1979–1986.

Kato, S., Nakamura, H., Naiki, M., Takeoka, Y. and Suehiro, S. (1991) "Suppression of acute experimental allergic encephalomyelitis by neurotropin: clinical, histopathologic, immunologic and immunohistochemical studies", *J. Neuroimmunol.* **35**, 237–245.

Lea, I.A., Richardson, R.T., Widgren, E.E. and O'Rand, M.G. (1996) "Cloning and sequencing of cDNAs encoding the human sperm protein, Sp17", *Biochim. Biophys. Acta* **1307**, 263–266.

Naiki, M., Suehiro, S., Imai, Y. and Osawa, T. (1989) "Immunomodulatory effects of neurotropin through the recovery of interleukin-2 production in autoimmune-prone (NZB/NZW) F1 mice", *Int. J. Immunopharmacol.* **11**, 663–671.

Naiki, M., Takeoka, Y., Kurimoto, Y., Matsuoka, T., Suehiro, S., Imai, Y., Osawa, T. and Gershwin, M.E. (1991) "Neurotropin inhibits experimental allergic encephalomyelitis (EAE) in Lewis rats", *Int. J. Immunopharmacol.* **13**, 235–243.

Nishikawa, K., Reddigari, S.R., Silverberg, M., Kuna, P.B., Yago, H., Nagaki, Y., Toyomaki, Y., Suehiro, S. and Kaplan, A.P. (1992) "Effect of neurotropin on the activation of the plasma kallikrein-kinin system", *Biochem. Pharmacol.* **43**, 1361–1369.

O'Rand, M.G. and Widgren, E.E. (1994) "Identification of sperm antigen targets for immunocontraception: B-cell epitope analysis of Sp17", *Reprod. Fertil. Dev.* **6**, 289–296.

O'Rand, M.G., Widgren, E.E. and Fisher, S.J. (1988) "Characterization of the rabbit sperm membrane autoantigen, RSA, as a lectin-like zona binding protein", *Dev. Biol.* **129**, 231–240.

Pan, M.R., Chuang, L.Y. and Hung, W.C. (2001) "Non-steroidal anti-inflammatory drugs inhibit matrix metalloproteinase- 2 expression via repression of transcription in lung cancer cells", *FEBS Lett.* **508**, 365–368.

Richardson, R.T., Yamasaki, N. and O'Rand, M.G. (1994) "Sequence of a rabbit sperm zona pellucida binding protein and localization during the acrosome reaction", *Dev. Biol.* **165**, 688–701.

Szabo, I.L., Pai, R., Soreghan, B., Jones, M.K., Baatar, D., Kawanaka, H. and Tarnawski, A.S. (2001) "NSAIDs inhibit the activation of egr-1 gene in microvascular endothelial cells. A key to inhibition of angiogenesis?", *J. Physiol. Paris* **95**, 379–383.

Yamasaki, N., Richardson, R.T. and O'Rand, M.G. (1995) "Expression of the rabbit sperm protein Sp17 in eos cells and interaction of recombinant Sp17 with the rabbit zona pellucida", *Molec. Reprod. Develop.* **40**, 48–55.

